# Treatment and Return to Sport in Athletes With Scaphoid Fractures: A Narrative Review

**DOI:** 10.7759/cureus.115689

**Published:** 2026-09-02

**Authors:** Yolanda Gutierrez, Paul Bonilla, Emmanuel Franco, Ronald Shaju, Alyssa Sepulveda, Nicholas Bonazza, Robert Schenck, Aamir Ahmad

**Affiliations:** 1 Medicine, University of Texas Rio Grande Valley School of Medicine, Edinburg, USA; 2 Medicine, University of Texas Rio Grande Valley School of Medicine, McAllen, USA; 3 Orthopedic Surgery, University of Texas Rio Grande Valley School of Medicine, Edinburg, USA; 4 Orthopedic Surgery, University of Arizona College of Medicine - Tucson, Tucson, USA

**Keywords:** athlete, nonunion, return to sport, review article, scaphoid fracture

## Abstract

Scaphoid fractures are the most common fracture of the carpal bone and frequently occur in athletic populations due to acute trauma or repetitive wrist loading. Management of these injuries in athletes is challenging because of the need to balance reliable fracture healing with timely return to sport. This narrative review summarizes current literature regarding treatment strategies, healing outcomes, and return-to-sport timelines in athletes with scaphoid fractures. We conducted a literature search using PubMed, Scopus, and CINAHL between July 2025 and January 2026. A total of 862 records were identified, with 255 duplicates removed. Following title, abstract, and full-text screening, nine clinical studies evaluating treatment outcomes and return to sport in athletes with scaphoid fractures were included. Additionally, three epidemiological studies describing the incidence and distribution of hand injuries in athletic populations were included for contextual analysis. Across the included studies, treatment strategies consisted of conservative management with immobilization or surgical fixation with percutaneous screw fixation or open reduction and internal fixation (ORIF). Return-to-sport timelines varied substantially according to treatment strategy, fracture characteristics, and chronicity, with some studies reporting earlier return following surgical fixation compared with conservative management. Available athlete-specific literature is limited primarily to case reports and small cohort studies, precluding definitive conclusions regarding treatment superiority. Further prospective research with standardized reporting of healing outcomes and return-to-sport criteria is needed to better guide management strategies for athletes with scaphoid fractures.

## Introduction and background

The scaphoid is the most commonly fractured carpal bone, accounting for approximately 60%-70% of all carpal fractures, and is typically injured following a fall onto an outstretched, dorsiflexed wrist [1]. Stress fractures are believed to be caused by repeated hyperextension of the wrist in sports that demand such movements, including but not limited to gymnastics, tennis, and diving [2]. This narrative literature review will focus primarily on return-to-sport rates, healing time, and long-term complications associated with nonoperative versus surgical management of scaphoid fractures in athletes.

The scaphoid bridges the proximal and distal carpal rows and articulates with the radius as well as the lunate, capitate, trapezium, and trapezoid, making it a critical stabilizer of wrist biomechanics [3]. Patients typically present with radial-sided wrist pain, tenderness within the anatomic snuffbox, decreased grip strength, and reduced wrist range of motion. Prompt diagnosis is essential because the scaphoid receives a predominantly retrograde blood supply, predisposing displaced or untreated fractures to nonunion, osteonecrosis, and scaphoid nonunion advanced collapse (SNAC) [4]. Further complicating the diagnosis of scaphoid fractures is the fact that initial radiographs often result in negative findings for scaphoid fractures [4]. Repeat imaging with radiographs, computed tomography (CT), or magnetic resonance imaging (MRI) is typically advocated in order to capture fractures that were clinically hidden earlier on [5].

Treatment of scaphoid fractures ranges from conservative management with casting and immobilization to surgical fixation via either percutaneous pinning or open reduction and internal fixation (ORIF) utilizing bone graft [6]. The latter is indicated for radiographically unstable fractures with a greater degree of displacement. Despite the treatment principles that have been established, management in athletes remains complex due to sport-specific demands and the desire to return to play promptly.

This narrative review aims to provide a summary of the existing literature regarding epidemiology, treatment strategies, return-to-sport timelines, healing outcomes, and complications. By summarizing the available evidence, we seek to provide a contextual understanding of management considerations in this unique population.

## Review

Methods of literature review

Study Design

This study was conducted as a narrative review using a structured literature search to qualitatively synthesize current evidence on the epidemiology, treatment strategies, healing outcomes, complications, and return-to-sport outcomes of scaphoid fractures in athletes. Although a systematic search strategy and structured study selection process were employed to improve transparency and reproducibility, the objective of this review was to provide a broad narrative synthesis of the literature rather than conduct a formal systematic review or meta-analysis. Because only publicly available literature was used, institutional review board approval was not required.

Literature Search Strategy

A comprehensive literature search was performed using PubMed, Scopus, and CINAHL. The search was conducted from July 2025 through January 2026 using Boolean search strategies that combined terms related to scaphoid fractures and athletic participation. The search strategy included the following: (scaphoid OR “scaphoid fracture” OR “scaphoid stress fracture” OR “carpal scaphoid”) AND (fracture OR fractures) AND (athlete OR athletic* OR sport* OR “sports participation” OR “return to sport” OR “return to play” OR “return to activity”).

Searches were limited to English-language studies involving human subjects. Reference lists of relevant articles were manually reviewed to identify additional relevant publications not captured during the initial database search.

Literature Selection

To improve consistency and transparency, all identified records were imported into Rayyan for organization, duplicate removal, and screening. Titles and abstracts were independently reviewed by multiple authors according to the objectives of this review, with disagreements resolved through discussion and consensus. Articles considered relevant underwent full-text review to determine their contribution to the narrative synthesis.

After removal of duplicates, 607 articles underwent title and abstract screening, followed by full-text review. The final review included nine clinical outcome studies examining treatment strategies, healing outcomes, complications, and return-to-sport outcomes in athletes with scaphoid fractures, in addition to three epidemiological studies that provided contextual information regarding the incidence and distribution of carpal injuries in athletic populations.

Data Synthesis

The included studies were reviewed qualitatively, with emphasis placed on epidemiology, treatment strategies, healing outcomes, complications, and return-to-sport outcomes. Owing to substantial heterogeneity in study design, patient populations, interventions, and reported outcomes, findings were synthesized narratively rather than quantitatively, and no formal meta-analysis was performed.

Formal risk-of-bias assessment tools were not applied because this review was intended as a narrative synthesis encompassing heterogeneous study designs, including observational studies, case series, and case reports. Instead, the strength and consistency of the available evidence were considered during interpretation of the findings.

Results

Overview of Included Studies

The literature included in this narrative review comprises a combination of epidemiological studies describing the incidence and distribution of scaphoid injuries in athletic populations and nine clinical outcome studies evaluating treatment strategies, healing times, and return-to-sport outcomes. The nine clinical outcome studies included a total of 89 patients and consisted of seven case reports and two retrospective cohort studies. There was substantial heterogeneity in fracture characteristics across the included studies, including fracture location, displacement, chronicity, and traumatic versus stress injury mechanisms. Athletic populations were also diverse and included participants in football, gymnastics, weightlifting, basketball, track and field, baseball, soccer, and Olympic-level sports.

Treatment Strategies and Return-to-Sport Outcomes

Clinical outcome studies consisted primarily of case reports and small retrospective cohorts evaluating conservative and surgical management of scaphoid fractures in athletes, as summarized in Table 1 [1,7-14]. Conservative treatment generally involved immobilization with casting or splinting, whereas surgical management included percutaneous or open screw fixation, with bone grafting used in selected cases. Reported return-to-sport timelines varied according to treatment and fracture characteristics. Surucu and Kehribar reported return to sport at seven weeks following percutaneous screw fixation compared with 11 weeks following cast immobilization. Among surgically managed athletes, return-to-sport timelines varied further according to fracture chronicity; Muramatsu et al. reported mean return to sport at 10.7 weeks for acute fractures, 14.0 weeks for fibrous unions, and 22.9 weeks for established nonunions [12]. Case reports demonstrated additional variability, particularly among stress fractures and delayed unions.

**Table 1:** Return-to-Sport Outcomes in Scaphoid Fractures in Athletes LIPUS: low-intensity pulsed ultrasound, ORIF: open reduction and internal fixation

Article	Study design	Sample size	Study population	Fracture type	Treatment modality	Time to return to sport	Healing time
Kohring et al. [7]	Case report	1	Shot put	Scaphoid stress fracture, incomplete, waist	Nonoperative: thumb spica splint	6 weeks	Radiographic resolution at 6 weeks
Surucu and Kehribar [8]	Retrospective comparative cohort	52	Reports a general inclusion of different athletes with no specific sport	Acute nondisplaced or minimally displaced scaphoid waist fractures	Percutaneous screw fixation versus cast immobilization	7 weeks (surgical) versus 11 weeks (cast)	8 weeks (surgical) versus 11 weeks (cast)
Nakamoto et al. [9]	Case report	1	Gymnastic	Scaphoid stress fracture (waist)	Percutaneous screw fixation	16 weeks	8 weeks
Mason et al. [10]	Case report	1	Weightlifting (bench press)	Acute nondisplaced scaphoid waist fracture	Conservative: cast → splint	Gradual return by 12 weeks; full unrestricted by ~6 months	Radiographic union ~3 months
Braun and Yelinek [11]	Case report	1	Collegiate basketball	Displaced scaphoid waist fracture (Herbert B2)	Nonoperative: thumb spica cast + LIPUS bone stimulator	Not assessed (season canceled due to COVID-19)	Progressive healing by ~3 months
Kohyama et al. [1]	Case report	1	Tennis	Scaphoid stress fracture, nondisplaced waist	Conservative: thumb spica cast (4 weeks) → wrist brace (8 weeks)	5 months (returned to competitive tennis)	Radiographic union at 3 months
Muramatsu et al. [12]	Retrospective cohort	30	Multiple sports (soccer, judo, basketball, baseball, track, boxing, rugby, tennis, etc.)/young competitive athletes	Acute fractures, fibrous union, and nonunion (Herbert A-D)	Open or percutaneous Herbert screw fixation ± iliac crest bone graft	Mean return to sport: 10.7 weeks (acute); 14 weeks (fibrous union); 22.9 weeks (nonunion)	Mean union: 9.2 weeks (acute); 8 weeks (fibrous union); 11.9 weeks (nonunion)
Matzkin and Singer [13]	Case report	1	Gymnastics/competitive youth	Scaphoid stress fracture, mid-waist, nondisplaced	Conservative: long-arm thumb spica cast (8 weeks) → short-arm splint (4 weeks)	6 months (returned to pre-injury gymnastic routine)	Radiographic union by 6 months
Reider et al. [14]	Case report	1	Weightlifting	Scaphoid waist fracture (delayed union, Herbert type C)	ORIF with Herbert screw + autologous radial bone graft	4 weeks cast → splint 3 months	Returned after radiographic healing (~4 months postoperative)

The criteria used to determine readiness for return to sport were inconsistently reported across the included studies. When described, return-to-sport decisions incorporated evidence of fracture healing and clinical recovery; however, standardized criteria for radiographic or CT-confirmed union, pain-free range of motion, grip strength, and sport-specific functional testing were not uniformly reported.

Epidemiology of Scaphoid Fractures in Athletes

Epidemiological data, as observed in Table 2 [15-17], demonstrated that scaphoid fractures represent a small subset of hand and wrist injuries in athletic populations. Surveillance-based studies involving collegiate athletes reported a scaphoid fracture rate of 0.11 per 10,000 athlete exposures, with higher rates observed in Division III athletes. Although the overall incidence was low, these findings demonstrate that scaphoid injuries occur across multiple sports and levels of athletic participation.

**Table 2:** Epidemiology of Hand Injuries in Athletes NCAA-ISP: National Collegiate Athletic Association Injury Surveillance Program, MRI: magnetic resonance imaging

Article	Study design	Athlete population	Sport(s)	Sample size	Reported epidemiological findings
Holoyda et al. [15]	Retrospective epidemiological analysis (NCAA-ISP)	Collegiate athletes (NCAA Divisions I-III)	Baseball, basketball, football, ice hockey, lacrosse, soccer, wrestling, gymnastics, softball, volleyball, field hockey	103,098 hand and wrist injuries	Scaphoid fracture rate 0.11 per 10,000 athlete exposures overall; highest in Division III
Alizai et al. [16]	Retrospective imaging-based epidemiological study	Elite Olympic athletes (Rio 2016)	Weightlifting, tennis, gymnastics, athletics, boxing, judo, rugby, cycling, shooting, others	11,274 athletes (25 underwent wrist MRI)	Scaphoid fractures identified in 32% (8/25) of athletes undergoing wrist MRI; scaphoid was the most commonly fractured carpal bone
Bennell et al. [17]	Prospective cohort study	Competitive athletes	Track and field	147 athletes (72 male and 75 female) followed for 12 months	Overall stress fracture incidence: 21% in female athletes and 7% in male athletes; incidence was not specific to scaphoid fractures

In elite Olympic athletes who underwent wrist magnetic resonance imaging (MRI), scaphoid fractures were identified in 32% of athletes undergoing wrist MRI and represented the most commonly fractured carpal bone in the cohort.

In addition, prospective cohort data in competitive track and field athletes demonstrated an overall stress fracture incidence of 21% in female athletes and 7% in male athletes over 12 months. Importantly, these data represent stress fractures across all anatomic locations and do not reflect the incidence of scaphoid stress fractures specifically. This study was included only to provide broader epidemiological context regarding stress-related injuries in athletic populations.

Discussion

In athletic populations, scaphoid stress fractures have been associated with repetitive loading and hyperextension of the wrist [18]. Understanding the sport-specific mechanisms of injury may further provide insight for clinicians in recognizing high-risk populations and guiding early diagnostic strategies. Consideration of complications between conservative and surgical management is a crucial step in deciding which approach fits best for which patient.

Importantly, scaphoid fractures in athletes represent a heterogeneous group of injuries, and treatment selection and return-to-sport timelines should be interpreted in the context of individual fracture characteristics. Fracture location is particularly relevant because proximal pole fractures have a more tenuous vascular supply and are associated with greater risks of delayed union and nonunion compared with waist and distal pole fractures. Similarly, fracture displacement, instability, chronicity, and the presence of established nonunion may favor operative management and prolong return to sport. Stress fractures also represent a distinct clinical entity from acute traumatic fractures, as they typically result from repetitive loading and may present with delayed diagnosis or established nonunion. Consequently, return-to-sport outcomes reported across studies should not be interpreted as directly interchangeable among all scaphoid fractures. These differences, as well as the heterogeneity of fracture characteristics reported in the available literature, limit direct comparison of treatment and return-to-sport outcomes across studies.

Surgical management, most commonly involving screw fixation, was reported to allow earlier mobilization and, in some cases, earlier return to sport compared with conservative treatment. However, return-to-sport timelines varied substantially according to fracture characteristics. Surucu and Kehribar reported return to sport at seven weeks following percutaneous fixation compared with 11 weeks following cast immobilization [8], while Muramatsu et al. reported progressively longer mean return-to-sport times for acute fractures (10.7 weeks), fibrous unions (14 weeks), and established nonunions (22.9 weeks) [12]. These findings illustrate the importance of fracture chronicity and healing status when interpreting return-to-sport outcomes. Athlete-specific literature has also reported earlier return following fixation; Rettig and Kollias reported an average return to sport of 5.8 weeks following Herbert screw fixation of acute middle-third scaphoid fractures in competitive athletes [19]. Differences in fracture location and stability, chronicity, athletic demands, postoperative protocols, and criteria used to determine return to sport likely contribute to variability in reported timelines. Given the predominance of case reports and small retrospective cohorts, these findings should be interpreted cautiously.

Among the conservatively managed cases included in this review, treatment failure was characterized by persistent or recurrent symptoms with absent or inadequate fracture healing, ultimately leading to surgical fixation [20,21]. This distinction is important because failure of conservative treatment should not be equated with the expected time required for fracture union. The SWIFFT randomized controlled trial by Dias et al. evaluated adults with scaphoid waist fractures displaced by 2 mm or less and supported initial cast immobilization, with suspected nonunion evaluated at 6-12 weeks and surgical fixation performed when nonunion was confirmed [22].

In the athlete-specific literature included in this review, a gymnast initially underwent conservative treatment with below-elbow casting and splinting [20]. After six months, imaging demonstrated apparent bony union and the athlete returned to full activity; however, wrist pain subsequently recurred, and a repeat scaphoid waist stress fracture was identified [20]. In another case, a 16-year-old acrobatic dancer treated conservatively demonstrated no evidence of fracture healing on follow-up imaging and subsequently underwent successful percutaneous screw fixation [21]. These cases illustrate circumstances in which conservative management was unsuccessful but should not be interpreted as evidence that surgical treatment is universally superior. Higher-level evidence from SWIFFT supports initial cast immobilization for undisplaced or minimally displaced scaphoid waist fractures, with surgical fixation reserved for confirmed nonunion [22]. Therefore, treatment failure should be considered in the context of fracture characteristics, progression toward union, persistent symptoms, and the functional demands of the athlete.

Beyond the reported time to return to sport, the objective criteria used to determine readiness for athletic participation are clinically important. Return-to-sport decisions should incorporate both fracture healing and functional recovery rather than rely solely on a predetermined postoperative or postinjury timeline. Assessment may include radiographic evidence of union, with CT used when confirmation of healing is necessary, as well as resolution of pain, restoration of wrist range of motion and grip strength, and the ability to perform sport-specific activities without symptoms. However, objective return-to-sport criteria were inconsistently reported among the studies included in this review, limiting the ability to establish standardized clearance criteria. Future studies should report both time to return to sport and the clinical, radiographic, and functional criteria used to determine readiness for return.

Higher-level evidence from non-athlete populations provides additional context regarding operative versus nonoperative management. In a randomized clinical trial of acute nondisplaced or minimally displaced scaphoid fractures, Vinnars et al. found that all fractures ultimately united and reported no significant long-term differences in subjective outcomes between operative and nonoperative treatment at a median follow-up of 10 years. However, scaphotrapezial osteoarthritis was significantly more prevalent in the operative group [23]. Similarly, a systematic review and meta-analysis of eight randomized controlled trials involving 419 patients found that surgical treatment favored some short-term outcomes, including functional outcomes, grip strength, time to union, and time off work, but found no significant differences in pain, range of motion, nonunion, or malunion. Complications were also more frequent in the surgical group, although this difference was not statistically significant [24]. These findings suggest that the potential short-term benefits of surgical fixation should be balanced against operative risks and the lack of clear long-term superiority, particularly for nondisplaced or minimally displaced fractures. Although these studies were not specific to athletes, they provide important higher-level evidence to contextualize the predominantly low-level athlete-specific literature included in this review.

Collectively, the available epidemiological literature suggests that although scaphoid fractures represent a relatively small proportion of athletic injuries overall, they occur across a broad range of sports and levels of competition. Their potential for delayed diagnosis, nonunion, and prolonged time away from sport underscores the importance of maintaining clinical suspicion for scaphoid injury in athletes presenting with persistent wrist pain.

This review has several strengths, including its focus on athletic populations and inclusion of a broad range of sports, further enhancing the applicability of findings across a diverse group of athletes. By incorporating epidemiological and clinical outcome data, this review provides a comprehensive overview of the burden and management considerations that are associated with scaphoid fractures in athletes.

Despite these strengths, several limitations should be acknowledged. Most included studies consisted of case reports and small retrospective cohort studies, limiting the overall level of evidence and generalizability of the findings. Substantial heterogeneity in fracture location, displacement, chronicity, presence of nonunion, and traumatic versus stress mechanisms further limited direct comparison of treatment and return-to-sport outcomes across studies. In addition, the criteria used to determine readiness for return to sport were inconsistently reported, including radiographic healing, clinical examination findings, functional recovery, and sport-specific testing. The absence of randomized controlled trials and standardized outcome reporting among the included athlete-specific studies limits the ability to draw firm conclusions regarding optimal management strategies. Finally, no formal risk-of-bias assessment tool was applied; evidence was instead evaluated qualitatively. These limitations highlight the need for prospective, sport-specific studies with standardized reporting of fracture characteristics, treatment protocols, healing outcomes, and objective return-to-sport criteria.

## Conclusions

Scaphoid fractures in athletes present unique management challenges due to the competing priorities of reliable fracture healing and timely return to sport. Athlete-specific literature suggests that surgical fixation may facilitate earlier return to sport, with reported return timelines typically ranging from approximately 7 weeks to 16 weeks following surgical treatment. However, higher-level evidence from general adult populations has not demonstrated clear long-term superiority of operative treatment for nondisplaced or minimally displaced fractures, emphasizing the need to balance potential short-term benefits against operative risks. Available athlete-specific evidence remains limited and largely consists of case reports and small cohort studies. Further prospective research with standardized reporting of healing outcomes and return-to-sport timelines is needed to guide treatment decisions in athletic populations better.
